# ACAT1/SOAT1 maintains adipogenic ability in preadipocytes by regulating cholesterol homeostasis

**DOI:** 10.1016/j.jlr.2024.100680

**Published:** 2024-10-30

**Authors:** Qing Liu, Xiaolin Wu, Wei Duan, Xiaohan Pan, Martin Wabitsch, Ming Lu, Jing Li, Li-Hao Huang, Zhangsen Zhou, Yuyan Zhu

**Affiliations:** 1 Department of Food Science and Nutrition, The Hong Kong Polytechnic University, Kowloon, Hung Hom, Hong Kong; 2 Department of Applied Biology and Chemical Technology, The Hong Kong Polytechnic University, Kowloon, Hung Hom, Hong Kong; 3 Shanghai Institute of Nutrition and Health, Chinese Academy of Sciences, Shanghai, China; 4 Division of Pediatric Endocrinology and Diabetes, Department of Pediatrics and Adolescent Medicine, University of Ulm, Ulm, Germany; 5 Department of Computing, The Hong Kong Polytechnic University, Kowloon, Hung Hom, Hong Kong; 6 Shanghai Key Laboratory of Metabolic Remodeling and Health, Institute of Metabolism and Integrative Biology, Liver Cancer Institute Zhongshan Hospital, Fudan University, Shanghai, China; 7 Research Institute for Future Food, The Hong Kong Polytechnic University, Kowloon, Hung Hom, Hong Kong; 8 The Hong Kong Polytechnic University Shenzhen Research Institute, The Hong Kong Polytechnic University, Shenzhen, China

**Keywords:** adipocytes, cholesterol/metabolism, cholesterol/trafficking, lipid rafts, nuclear receptors/SREBP, PPARγ, cholesteryl ester

## Abstract

Maintaining cholesterol homeostasis is critical for preserving adipocyte function during the progression of obesity. Despite this, the regulatory role of cholesterol esterification in governing adipocyte expandability has been understudied. Acyl-coenzyme A (CoA):cholesterol acyltransferase/Sterol O-acyltransferase 1 (ACAT1/SOAT1) is the dominant enzyme to synthesize cholesteryl ester in most tissues. Our previous study demonstrated that knockdown of either ACAT1 or ACAT2 impaired adipogenesis. However, the underlying mechanism of how ACAT1 mediates adipogenesis remains unclear. Here, we reported that ACAT1 is the dominant isoform in white adipose tissue of both humans and mice, and knocking out ACAT1 reduced fat mass in mice. Furthermore, ACAT1-deficiency inhibited the early stage of adipogenesis via attenuating PPARγ pathway. Mechanistically, ACAT1 deficiency inhibited SREBP2-mediated cholesterol uptake and thus reduced intracellular and plasma membrane cholesterol levels during adipogenesis. Replenishing cholesterol could rescue adipogenic master gene–*Pparγ*′s—transcription in ACAT1-deficient cells during adipogenesis. Finally, overexpression of catalytically functional ACAT1, not the catalytic-dead ACAT1, rescued cholesterol levels and efficiently rescued the transcription of PPARγ as well as the adipogenesis in ACAT1-deficient preadipocytes. In summary, our study revealed the indispensable role of ACAT1 in adipogenesis via regulating intracellular cholesterol homeostasis.

The bioprocess of cholesterol esterification has been considered as a critical pathway to regulate cholesterol homeostasis (1), contributing to the detoxification of the over-accumulated free cholesterol (FC) intracellularly, lipid droplet (LD) biogenesis in epithelial cells, hepatocytes, foam cells, and cancer cells (2), as well as the regulation of antitumor response of CD8^+^ T cells (3). Interestingly, LD-richest adipocytes are low in cholesteryl ester (CE), thus, the bioprocess of cholesterol esterification has been understudied.

Cholesterol esterification is so far known to be mediated by acyl-CoA: cholesterol acyltransferase (ACAT) [as known as sterol O-acyltransferase (SOAT)], an endoplasmic reticulum (ER)-localized protein, esterifies CE from FC and fatty acids with the help of ATP and coenzyme A (4). Two isoforms, ACAT1 and ACAT2, have been identified. ACAT1 is expressed widely in both humans and mice, while ACAT2 exhibits high expression in human intestinal enterocytes (5) and murine hepatocytes (6, 7). In the past decades, ACAT1 has emerged as a promising target for the treatment of various types of cancer, such as pancreatic cancer (8), gastric cancer (9) and hepatocellular carcinoma (10).

The cholesterol content in adipose tissue increases during white adipose tissue (WAT) expansion (11). Obese humans were estimated to store one-third to half of the body cholesterol in WAT, with lean humans about 25% (12). When looking into the adipocyte per se, cholesterol is mainly located in the plasma membrane (PM) and LD membrane (13), and adipocyte size is positively related to the contents of triglycerides (TG) and FC during obesity progression (14). Notably, compared to smaller adipocytes, larger adipocytes contain lower FC in the PM (15), suggesting that the intracellular distribution of FC is associated with the adipocyte expansion process. Given that PM-localized FC is important for mediating extracellular stimuli’s signaling via FC-enriched lipid rafts (16), the decrease in PM cholesterol levels during adipocyte hypertrophy may affect WAT expansion. Furthermore, given that intracellular cholesterol homeostasis is critical for maintaining PM fluidity, signaling transduction, and hormonal production (1), it is important to investigate the role of cholesterol homeostasis in adipocytes. Therefore, cholesterol esterification, together with cholesterol uptake, transport, de novo synthesis, metabolism, and efflux (17), is expected to impact WAT expansion as a key regulator of cholesterol homeostasis.

Previously, we found that either ACAT1 or ACAT2 deficiency attenuated adipogenic ability in murine 3T3-L1 preadipocytes, which is partially attributed to the reduced expressions of sterol regulatory element binding protein 1 (SREBP1)’s downstream lipogenic genes (18). However, the main ACAT isoform in WAT and how ACAT1 regulates adipogenesis remain unclear.

In the present study, we found that ACAT1 was the primary isoform in WAT of mice and humans. Furthermore, ACAT1-deficiency inhibited adipogenesis at the early stage via attenuating *Pparγ* transcription. Mechanistically, ACAT1 deficiency suppressed the expression of SREBP2’s downstream genes involved in cholesterol uptake and reduced the increase in PM-localized cholesterol content during adipogenesis. This contributed to the impaired induction of *Pparγ* transcription in response to adipogenic stimulation. Collectively, our results demonstrated that ACAT1 helps maintain adipogenic ability in preadipocytes, partially by modifying cholesterol uptake and distribution, and that the role of ACAT1 in adipogenesis can be integrated into the PPARγ-mediated adipogenesis network, underscoring the significant role of cholesterol esterification in the expansion of adipose tissue.

## Materials and methods

### Animal model

The mouse models used in this study were on the C57BL/6J background. Experiments were performed with age-matched male littermates at the age of 8–10 weeks on a standard chow diet in a temperature-controlled room on a 12-h light/dark cycle. They were fed with a standard chow diet (PicoLab Rodent Diet 20) and given sterile water ad libitum. *Acat1* ^*flox/flox*^ mice were constructed by inserting two loxp sites covering ACAT1 exon 14 [kindly provided by Dr Ta-Yuan CHANG (19)]. ACAT1 global knockout mice were generated by homologous recombination which resulted in a deletion of the 2kb sequence in the genome [originally generated by Dr Robert V. Farese, Jr (20) and kindly provided by Dr Ta-Yuan Chang (19)]. The animals presented a healthy status, and male mice were used for all experiments. ACAT1 global knockout mice experiments were performed at the Shanghai Institute of Nutrition and Health, Chinese Academy of Sciences, and all animals were maintained and used in accordance with the guidelines of the Institutional Animal Care and Use Committee. Other mice-related experiments were performed at The Hong Kong Polytechnic University with ethics approved by the Centralized Animal Facilities (CAF).

### Cell culture and treatment

3T3-L1 murine preadipocytes (ATCC #CL-173) were obtained and cultured in DMEM medium (Gibco #11995065) supplemented with 10% FBS and 100 IU/ml penicillin/streptomycin. SGBS cells, generously provided by Prof. Martin Wabitsch, were cultured following previously described protocols (21, 22). All cells were cultured at 37°C in a humidified atmosphere with 5% CO_2_.

#### Stromal vascular fraction (SVF) cell isolation

Ing-WAT from male *Acat1* ^*flox/flox*^ mice or WT mice aged 4–6 weeks on a chow diet were minced with scissors. The isolation medium was prepared by adding 1 mg/ml collagenase type II (17101015, Thermo) and 2.4 IU/ml Dispase II (D4693, Sigma-Aldrich) in HBSS buffer. The minced fat pads were digested using a digestion solution and incubated in a shaking water bath at 37°C for 30–60 min. The suspensions were then filtered through a 100 μm cell strainer (Falcon), and pre-warmed PBS buffer was added. After centrifugation at 1000 × g for 5 min, the cell pellets were collected from the bottom of the tube and cultured with DMEM.

#### Adipocyte differentiation

For 3T3-L1 and Ing-WAT-isolated SVFs, the adipocyte differentiation protocol was slightly modified from a previous study (18). Briefly, after the cells reached 100% confluence for two days, the adipogenic cocktail (10 μg/ml insulin (I6634, Sigma-Aldrich), 0.5 mM 1-methyl-3-isobutylxanthine (I7018, Sigma-Aldrich), 1 μM dexamethasone (D4902, Sigma-Aldrich) and 0.1 μM rosiglitazone (R2408, Sigma-Aldrich)) were loaded for 2 days. Then cells were incubated with 10 μg/ml insulin until harvest day. LD were stained with Oil red O (ORO) (O1391, Sigma-Aldrich), and were visualized with the help of optical microscopy (Olympus CKX41). The lipids stained with ORO were eluted using 100% isopropanol and the absorbance was measured at 500 nm.

### Plasmids

To generate the SRE-luciferase plasmid, four copies of the sterol regulatory element (SRE, ATCACGTG) from the pGMSREBP (Yeasen) were inserted into a pNL2.1-Nluc (Promega) luciferase vector. RFP labeled Domain 4 plasmid were kindly provided by Dr Deng Yongqiang (Southern Medical University) and the sequence was listed in supplemental Table S2.

### Luciferase assay

shCTRL or shACAT1 cells were transfected with pGL4.2-SRE-Luc2 plasmids. After a 3-day differentiation period, the luciferase signal was measured by using the Dual-Luciferase® Reporter Assay System (Promega E1910) according to the methods previously described (23). Luminescence was determined by the microplate reader (CLARIOstar).

### Virus transduction

Lentivirus (Lent-U6-GFPshACAT1-puro) containing mouse ACAT1 (NM-009230.3) shRNA or control shRNA was purchased from VigeneBio. The shRNA sequence for ACAT1 was 5′-GCAAGAGTTCTCACCCATTGA-3′, and the scrambled sequence of shCTRL was 5′- TTCTCCGAACGTGTCACGT- 3’. Lentivirus was employed to infect 3T3-L1 preadipocytes, and then the cells were selected with 2 μg/ml of puromycin for 3 days to generate a stable cell line.

Adenoviruses carrying the Cre gene (Ad5-CMV-Cre-mCMV -copGFP) or empty adenovirus (Ad5-CMV-copGFP) were purchased from VigeneBio (Jinan). Adenoviruses expressing human ACAT1 (pADM-CMV-ACAT1-mCherry), catalytical dead ACAT1 (H460A, refer to the previous study (24)) or control sequence (pADM-CMV-mCherry) were purchased from VigeneBio. Adenoviruses were added to 3T3-L1 preadipocytes, and primary preadipocytes and incubated for 6–12h.

### RNA sequencing (RNA-seq)

Total RNA was isolated from shCTRL or shACAT1 3T3-L1 adipocytes (n = 2) using Trizol reagent. RNA-seq was performed by BGI Tech Solutions (Hong Kong) Co., Limited. In this study, differentially expressed genes (DEGs) were determined using a threshold of *P* < 0.05 and fold change > 1.5. The identified DEGs were then subjected to analysis for enriched biological processes using the top GO package. Enriched pathways were identified using Fisher's exact test with a significance level of *P* < 0.05 for KEGG pathway analysis. GSEA software was utilized to generate GSEA enrichment plots, and default parameters were applied for the analysis.

### Gene expression analysis

Total RNA was extracted with TRIzol (TR 118, Millipore) method and then reverse-transcribed into cDNA with the PrimeScript™ RT Master Mix (RR036A, Takara), following the manufacturer’s instructions. Quantification of PCR amplification products was performed by using the Applied Biosystems QuantStudio 7 Flex Real-Time PCR System (Thermo) and SYBR (RR420A, Takara). The mRNA expression levels of interest genes were normalized to that of β-actin or GAPDH with the delta-delta Ct method. The primer sequences for qPCR are shown in supplementary Table S3.

### Western blotting

The proteins were extracted by RIPA lysis buffer (P0013B, Beyotime) supplemented with an inhibitor cocktail (HY-K0021, MedChem Express). Protein concentrations were determined using the BCA Protein Assay Kit (23225, Thermo Fisher Scientific). Equal amounts of protein were loaded onto 12% SDS-PAGE gels, followed by transfer to PVDF membranes (Millipore), as previously described (25). The primary antibodies against ACAT1, GLUT4, and SREBP2 were purchased from Abcam, and the primary antibodies against PI3K, P-PI3K, P-AKT (Ser-473), P-AKT (Ser-308) from Cell Signaling Technology. The primary antibodies against GAPDH, Caveolin-1 were purchased from Thermo. Primary antibodies were all diluted by 1:1000. Secondary antibodies of anti-mouse (1:10,000) or anti-rabbit (1:10,000) were purchased from Thermo. The membranes were processed by ECL (32106, Thermo) and delivered to ChemiDoc Imaging System (Bio-Rad) to capture pictures, followed by ImageJ analysis. The quantification of specific protein level was normalized to GAPDH.

### Immunofluorescence staining

Cells grown on glass coverslips in a 12-well plate were fixed with 10% (v/v) neutral buffered formalin for 20 min at room temperature (RT). After rinsing with PBS, the samples were permeabilized with 0.1% (v/v) Triton X-100 for 5 min. Following another round of PBS rinsing, the samples were incubated with the primary antibody at a 1:100 dilution at 4°C overnight. They were then incubated with a secondary antibody at a 1:500 dilution at RT for 1 h, following three washes with PBS. The primary antibodies against caveolin-1 were purchased from Thermo. Hoechst 33342 (H1399, Thermo) was applied to stain nuclei following the standard procedure. Finally, the coverslips were mounted on slides using an antifade solution (#S36936, Thermo). Confocal microscopy was performed using a Leica TCS SPE Confocal Microscope (Leica, Germany) with a 63X/1.4 oil immersion objective unless stated otherwise.

### Cholesterol and cholesteryl ester measurement

The lipids were extracted by mixing hexane (Sigma) and isopropanol (Sigma) (3:2) for 30 min from adipocytes or adipose tissue, and then dried through nitrogen and further diluted with 1x reaction buffer. The FC and CE levels were measured using the Amplex Red cholesterol assay kit (A12216, Thermo) according to the manufacturer’s protocol. The cholesterol contents were normalized to the cell number or tissue weight.

The measurement of PM and intracellular cholesterol content was conducted using a cholesterol oxidation-based method, following the previously described protocol (3). The measurement of PM cholesterol was derived by subtracting the intracellular cholesterol content from the total cellular cholesterol.

### Modulation of the cholesterol level by methyl-beta-cyclodextrin (MβCD) and MβCD-coated cholesterol

To deplete lipids from serum, fumed silica (Sigma, S5130, 20 g/L) was added to FBS, followed by overnight incubation, centrifugation, and filtration (26). For cholesterol depletion from the PM, adipocytes were treated with 5 mM MβCD for 15 min at 37°C, followed by three washes with PBS. To incorporate cholesterol into the PM, adipocytes were exposed to a culture medium containing 20 μg/ml MβCD-coated cholesterol and incubated at 37°C for 15 min (3).

### Glucose uptake

3T3-L1 cells were starved by treating them with KRPH buffer for 40 min on the day of harvest and subsequently incubated with or without 1 μM insulin for 20 min. Subsequently, the wells were treated with 10 mM 2-DG to measure glucose uptake using the Glucose Uptake Colorimetric Assay Kit (#K676, BioVision), following the manufacturer's instructions.

### Statistical analysis

All data were expressed as means ± S.E.M. as indicated in the figure legends. The statistical significance for comparisons between control and treatment groups was determined by a two-tailed student's *t* test, one-way or two-way ANOVA followed by Tukey’s test (for comparison of three or more groups), as indicated in the figure legends, by using GraphPad Prism software. Statistical significance is reported when *P* value < 0.05.

## Results

### ACAT1 is the dominant isoform in WAT from humans and mice

**Fig.1:**
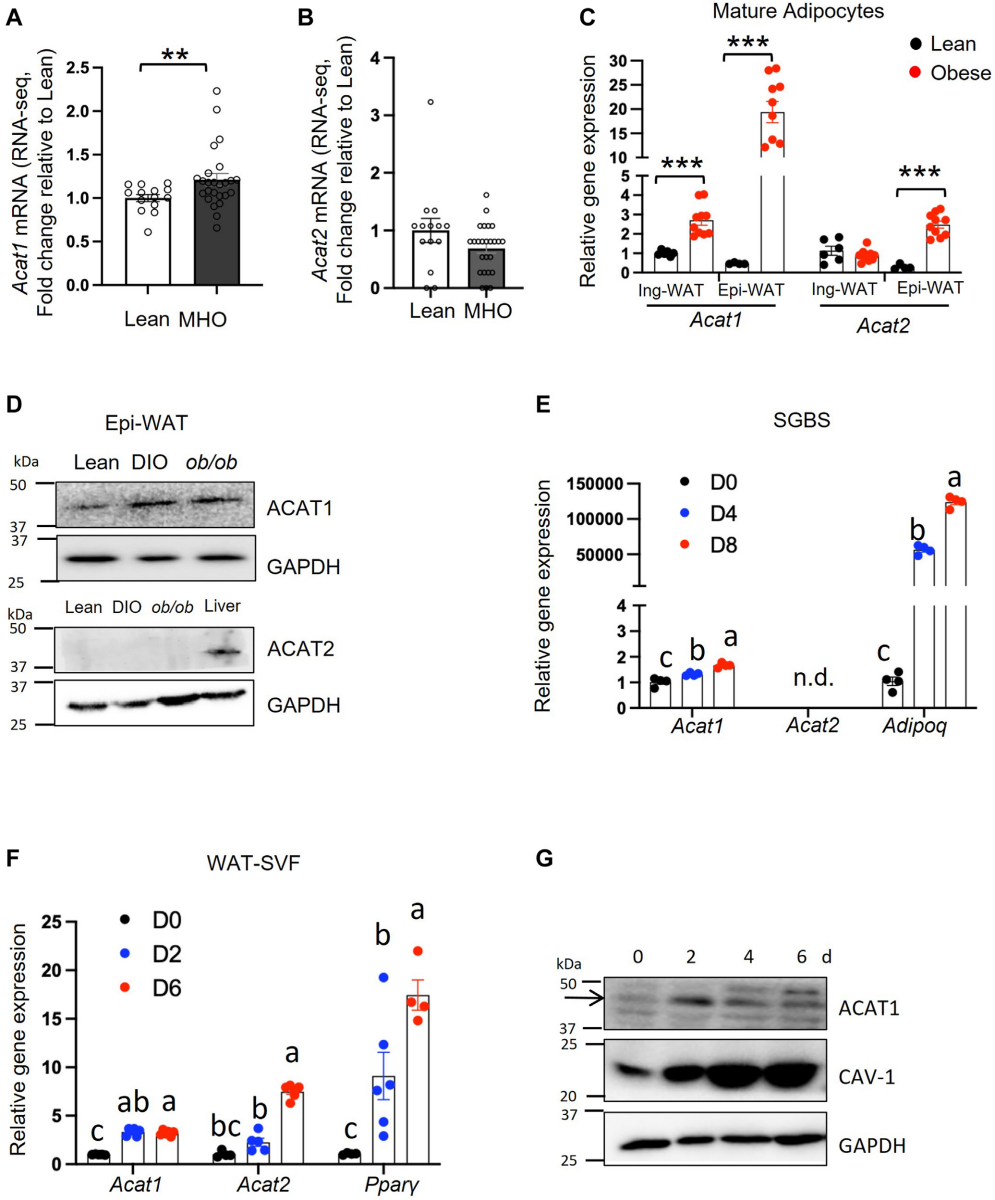
ACAT1 is the dominant isoform in WAT from humans and mice. The relative mRNA levels of (A) ACAT1 and (B) ACAT2 in subcutaneous abdominal adipose tissue in the Lean-normal (n = 14) and Metabolic Healthy Obese (MHO; n = 25) subjects, based on RNA-seq data analysis. C: *Acat1* and *Acat2* mRNA levels in mature adipocytes isolated from epidydimal WAT (Epi-WAT) or inguinal WAT (Ing-WAT) of lean and high-fat diet-induced obese mice, respectively. D: ACAT1 and ACAT2 protein levels in Epi-WAT of lean, high-fat diet-induced DIO mice and *ob/ob* mice by western-blot (WB). E: SGBS preadipocytes were differentiated and collected at different time points during adipogenesis for the analysis of indicated genes using qPCR. White adipose stromal vascular fraction (SVF) differentiated adipocytes were collected at different time points during adipogenesis for the analysis of indicated genes using qPCR (F) and WB (G). Data is presented as Mean ± SEM (n = 3–5) and analyzed by student *t* test (A–C) or two-way ANOVA followed by Tukey’s test (E, F). ∗∗*P* < 0.01, ∗∗∗*P* < 0.001. Different letters indicate statistically significant difference (*P* < 0.05).

To identify the main isoform of ACAT in WAT, we re-analyzed *Soat1* and *Soat2* (the gene name of *Acat1/2* in RNA-seq) expressions from the published RNA-seq data of subcutaneous WAT (sWAT) in humans. We found that *Acat1/Soat1* mRNA in sWAT from lean people was significantly lower than that from metabolic healthy obese (MHO) ones [Fig. 1A, data from (27); supplemental Fig. S1A, data from (28); supplemental Fig. S1C, data from (29)]. In contrast, *Acat2*/*Soat2* mRNA in sWAT was extremely low and had little difference between lean and MHO subjects [Fig. 1B, data from (27); supplemental Fig. S1B data from (28); supplemental Fig. S1D, data from (29)]. Therefore, ACAT1/SOAT1, instead of ACAT2/SOAT2, is the dominant isoform of ACAT in human sWAT.

Considering the cellular heterogeneity in WAT, we further examined the expression of ACAT1/2 in mature adipocytes in mice. We isolated mature adipocytes from WAT in 20-week-old lean and diet-induced obese (DIO) mice (male, C57BL/6J, on a high-fat diet for 14 weeks), respectively. Both *Acat1/Soat1* and *Acat2/Soat2* expressed at the transcriptional level in mature adipocytes from either epidydimal WAT (Epi-WAT) or inguinal WAT (Ing-WAT) in lean and obese mice, with *Acat1* mRNA higher in obese than lean mice (Fig. 1C). However, only ACAT1 protein, instead of ACAT2, was detected in the Epi-WAT of mice (Fig. 1D). Supportively, globally knocking out ACAT1 significantly reduced the amount of Ing-WAT and Epi-WAT (supplemental Fig. S1E, F), although with similar body weight (supplemental Fig. S1G) and without ACAT2 compensation (supplemental Fig. S1H, I), as compared to wild type (WT) mice. Collectively, these data suggested that ACAT1 is the dominant isoform in the WAT of mice.

One of the main functions of WAT is to store extra energy by increasing adipocyte number (adipogenesis) and size (adipocyte hypertrophy). To identify the expression of ACAT1/2 during adipogenesis, we employed cell models from both murine and human sources. As expected, *Acat1* mRNA was upregulated upon adipogenic stimulation in human SGBS preadipocytes without *Acat2* inducement (Fig. 1E). In two murine cell models: mature adipocytes differentiated from mouse white adipose SVF (Fig. 1F, G) and murine 3T3-L1 preadipocyte cell line (supplemental Fig. S1J–L), ACAT1 was induced at both mRNA and protein levels during adipogenesis (Fig. 1F, G, supplemental Fig. S1J–L). Furthermore, the level of CE and CE: FC were also upregulated during adipogenesis in 3T3-L1 differentiated adipocytes (supplemental Fig. S1M–O). Collectively, these results demonstrated a positive association between ACAT1 and adipogenesis both in murine and human preadipocytes.

### ACAT1 deficiency inhibited adipogenesis at the early stage via attenuating PPARγ pathway

**Fig. 2:**
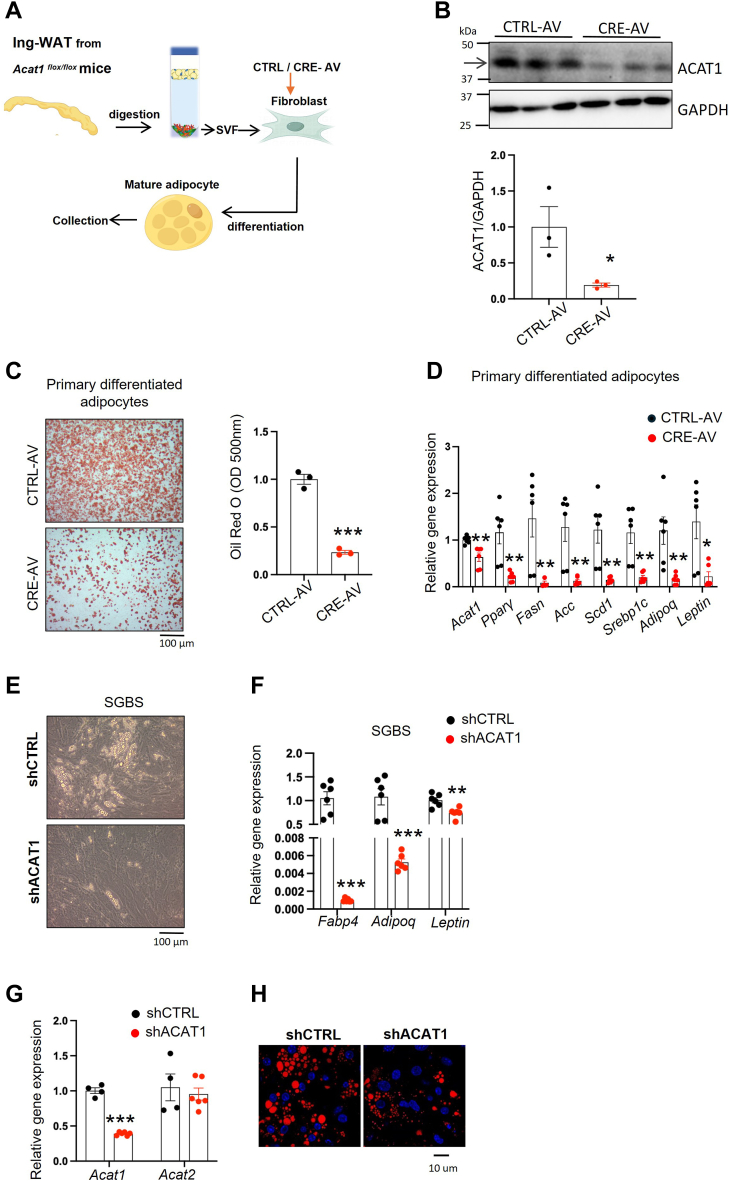
ACAT1 deficiency in preadipocytes impairs adipogenesis. A: Schematic of knockout (KO) of ACAT1 in WAT-SVF with adenovirus (AV) and the subsequent adipogenesis. Ing-WAT derived SVF cells from *Acat1*^flox/flox^ mice were infected with CTRL-AV or CRE-AV (adenovirus expressing Cre recombinase) to generate either CTRL cells or ACAT1-KO cells, respectively. The ACAT1 protein levels were determined by Western blot (B). After differentiation, the adipocytes were stained with Oil Red O (C), and mRNA level of genes involved in adipogenic transcription program and TG synthesis were quantified by qPCR (D). ACAT1 was knocked down with lentivirus-mediated shRNA in human SGBS preadipocytes, which were further differentiated to mature adipocytes, and lipid droplets (LDs) were captured under a light microscope (E), and mRNA levels were quantified with qPCR (F). In 3T3-L1 cell line, shCTRL and shSOAT1 preadipocytes were collected to quantify for the mRNA levels of indicated genes with qPCR (G), and after differentiation, the LDs were stained with Nile red (H). Data is presented as Mean ± SEM (n = 3–5) and analyzed by student *t* test. ∗*P* < 0.05, ∗∗*P* < 0.01, ∗∗∗*P* < 0.001.

Previously, we have demonstrated that inhibiting ACAT1 or knocking down *Acat1* would attenuate adipogenesis in murine 3T3-L1 preadipocytes (30). However, the underlying mechanism remains unclear. Firstly, given the limitation of 3T3-L1 preadipocytes, we employed two other models to validate the phenotype: primary differentiated adipocytes and human preadipocyte cell line. We isolated SVF from Ing-WAT of *Acat1* ^*flox/flox*^ mice and applied Cre-expressing adenovirus to knockout *Acat1* at the preadipocyte stage (Fig. 2A), with the knockout efficiency of 80% at the protein level (Fig. 2B). In agreement with our previous findings, knocking out *Acat1* at the preadipocyte stage significantly attenuated adipogenesis, as determined by the reduced lipid content (Fig. 2C) and adipogenic-related gene expression (*Pparγ, Fasn, Acc, Scd1*) (Fig. 2D). Furthermore, *Acat1*’s role in maintaining adipogenesis was also confirmed in human SGBS preadipocytes (Fig. 2E, F), without *Acat2* compensation (undetected value). This phenotype was consistent with that in 3T3-L1 preadipocytes, where shRNA-mediated knockdown of ACAT1 resulted in impaired adipogenesis (without a significant compensatory increase in *Acat2* mRNA) (Fig. 2G, H).

**Fig. 3:**
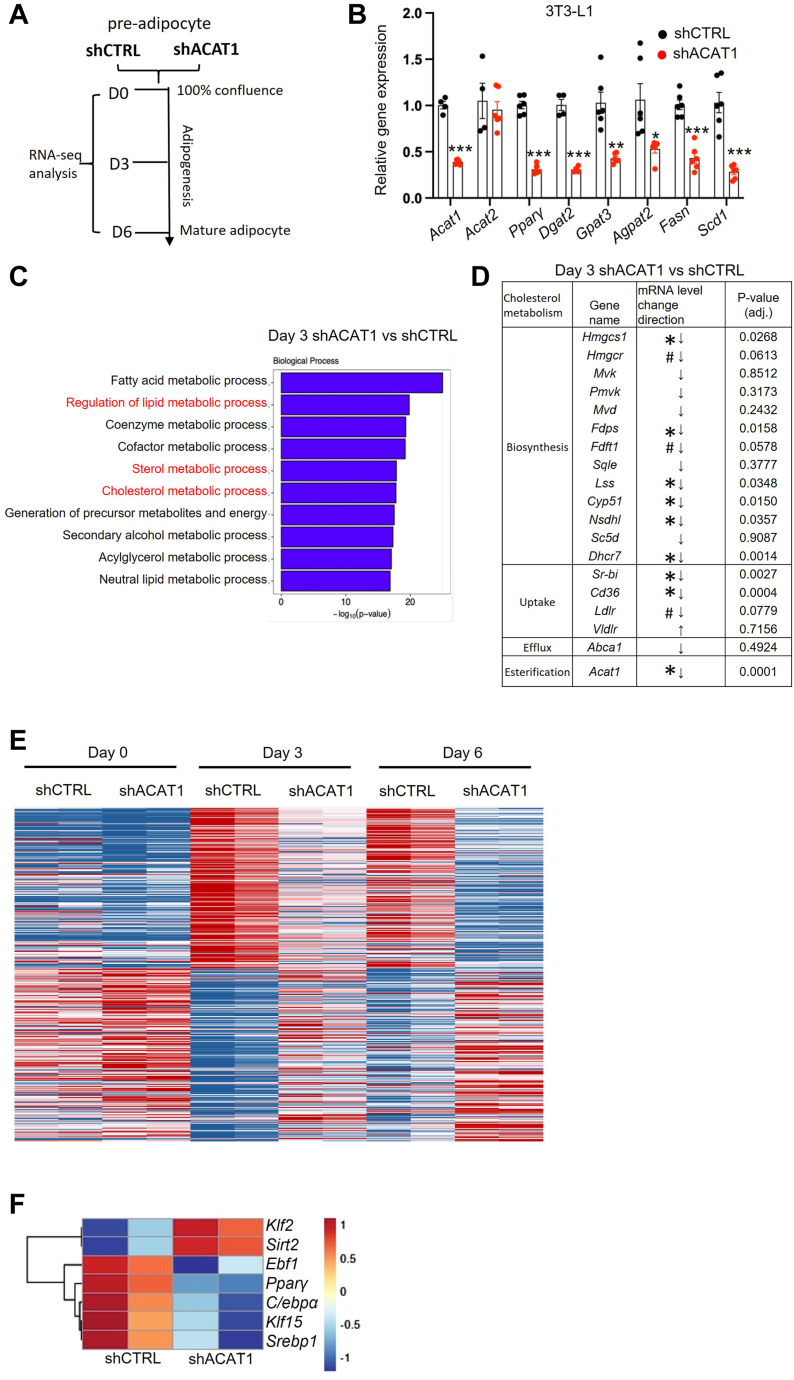
ACAT1 deficiency suppresses adipogenesis at the early stage. A: Schematic of cell samples for RNA-sequencing. B: in 3T3-L1 cell line, mRNA levels of adipogenic genes in shCTRL and shACAT1 adipocytes upon 3 days of differentiation were quantified by qPCR. C: top 10 downregulated signaling pathways analyzed by the biological process in 3T3-L1 differentiated shACAT1 versus shCTRL cells in Day 3. D: the expression of genes for cholesterol metabolism in 3T3-L1 shACAT1 versus shCTRL cells after 3 days’ adipogenic stimulation. E: the heatmap of genes with significant differences between shACAT1 versus shCTRL cells at Days 0, 3, and 6, respectively. F: the heatmap analysis of the key representative genes in the early stage of adipogenesis was shown to compare shACAT1 and shCTRL cells on Day 3. Data is presented as Mean ± SEM (n = 3–5) and analyzed by student *t* test. #*P* < 0.1, ∗*P* < 0.05, ∗∗*P* < 0.01, ∗∗∗*P* < 0.001.

To map the transcriptional changes caused by ACAT1 deficiency during adipogenesis, we generated shCTRL and shACAT1 preadipocytes in murine 3T3-L1 cells with lentivirus-mediated shRNA-based interference system and performed RNA sequencing (RNA-seq) analysis on shCTRL and shACAT1 cells at day 0, day 3 and day 6 post adipogenic stimulation, respectively (Fig. 3A). The deficiency of ACAT1 attenuated adipogenesis as characterized by the lower transcripts of genes for TG synthesis (*Pparγ, Dgat2*), lipid accumulation (*Gpat3, Agpat2*) and fatty acid synthesis (*Fasn, Scd1*) after 3 days’ adipogenic stimulation (Fig. 3B), as compared to shCTRL cells. The analysis of biological processes showed that the lack of ACAT1 attenuated the transcriptional inducement in genes associated with metabolic processes involving fatty acid and cholesterol metabolism during the early stage of adipogenesis (Fig. 3C). The majority of genes in cholesterol metabolism were downregulated in ACAT1-deficient adipocytes (Fig. 3D). This reduction was even more pronounced during the later stage of adipogenesis (Fig. 3E).

Adipogenesis is predominantly regulated by turning on or off a cascade of transcriptional regulators in the early stage (31). Accordingly, the key transcriptional factors that promote adipogenesis, including *Pparγ* (32), *C/ebpα* (33), *Klf15* (34), *Ebf1* (35), and *Srebp1* (36), were less responsive to the adipogenic cocktail in shACAT1 preadipocytes than shCTRL ones (Fig. 3F). In contrast, the genes that negatively regulate adipogenesis, such as *Klf2* (37) and *Sirt2* (38), were either upregulated or failed to be downregulated during adipogenesis in shACAT1 preadipocytes, as compared to shCTRL ones (Fig. 3F). Collectively, ACAT1-deficiency suppressed the inducement of adipogenic transcriptional cascade up on adipogenic stimulation at the early stage of adipogenesis in vitro.

**Fig. 4:**
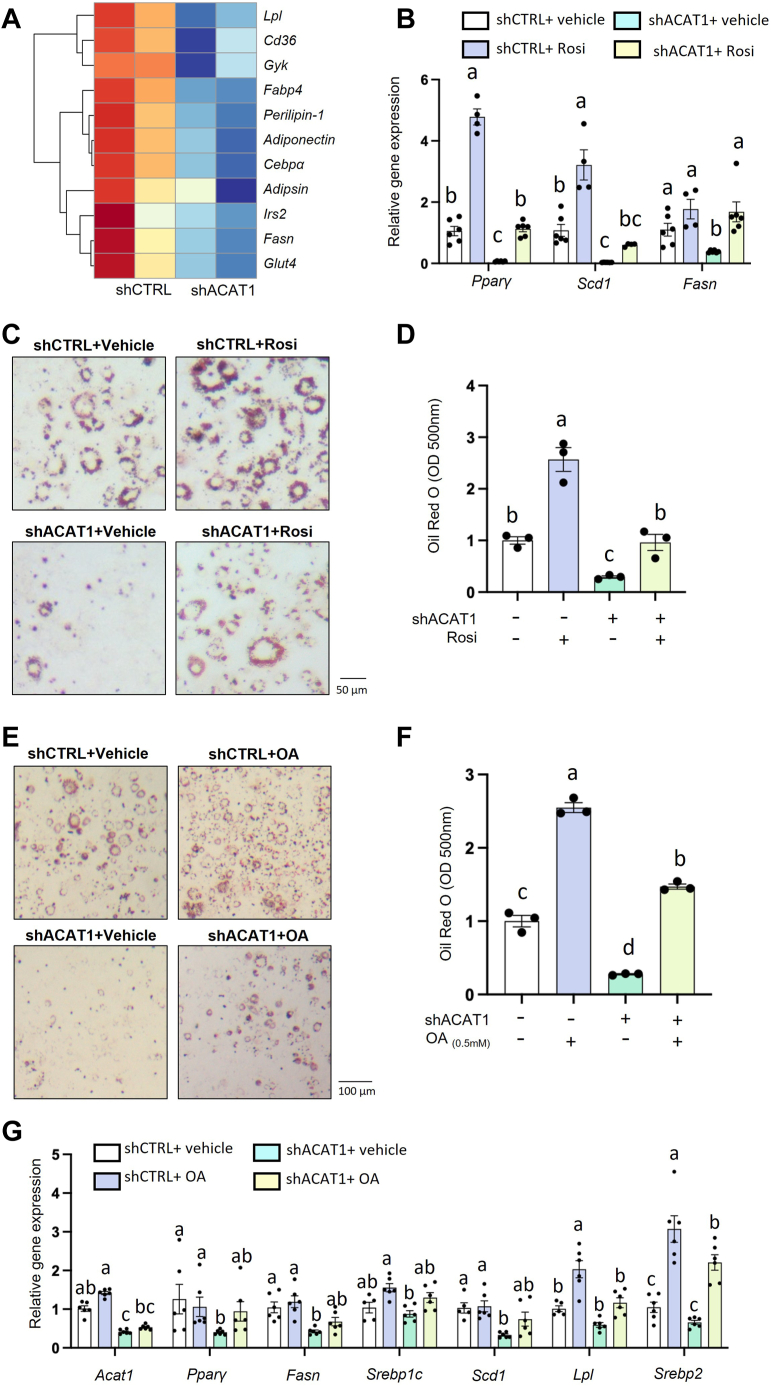
Enhanced PPARγ pathway rescues adipogenesis in ACAT1 deficient cells. A: The heatmap analysis of the PPARγ targeted genes was performed in 3T3-L1 differentiated shACAT1 versus shCTRL cells after 3 days of differentiation. Rosiglitazone (2 μM) was added in shCTRL and shACAT1 (3T3-L1) cells at the early stage of adipogenesis (day 0–2), and mature adipocytes in each group were collected for quantifying (B) mRNA levels of adipogenic genes, as well as (C, D) lipid content with ORO. Oleic acid (0.5 mM) was supplemented in shCTRL and shACAT1 (3T3-L1) cells during the early stage of adipogenesis, mature adipocytes in each group were collected for quantifying (E, F) lipid content with ORO, and (G) mRNA levels of genes with qPCR. Data is presented as Mean ± SEM (n = 3–5) and analyzed by two-way ANOVA followed by Tukey’s test. Different letters indicate statistically significant difference (*P* < 0.05).

Considering that *Pparγ* is a “master” transcriptional factor for adipogenesis at the early stage and that the downstream target genes of *Pparγ* were all decreased in the shACAT1 group compared with shCTRL (Fig. 4A), we hypothesized that ACAT1 deficiency impaired adipogenesis via PPARγ pathway at the early stage of adipogenesis. To test it, we loaded the PPARγ agonist, rosiglitazone (Rosi), during the first two days of adipogenesis and found that the transcriptional levels of *Pparγ* and its downstream genes (*Scd1, Fasn*) (Fig. 4B), as well as the accumulated lipid level (Fig. 4C, D), could be fully rescued in shACAT1 preadipocytes. Additionally, given that fatty acids and its derivatives are PPARγ ligands (39) and the genes for fatty acid synthesis (like *Fasn*, *Scd1*) were significantly lower in shACAT1 cells than shCTRL ones at the early adipogenic stage (Fig. 3B), the reduction of de novo fatty acid synthesis should also contribute to the impaired PPARγ pathway in shACAT1 cells. Accordingly, supplementation with oleic acid (OA), as a PPARγ ligand and a substrate for TG synthesis, could successfully enhance lipid accumulation ability in both shCTRL and shACAT1 preadipocytes during adipogenesis (Fig. 4E, F), with the increment in the mRNA levels of associated genes (*Pparγ, Srebp1c, Scd1, and Fasn*) (Fig. 4G). Therefore, the attenuation of adipogenic ability at the early stage of adipogenesis in ACAT1-deficient preadipocytes was, at least partially, PPARγ-dependent.

### ACAT1-deficiency attenuated *Pparγ* transcription, partially by reducing PM-cholesterol

Given that cholesterol-enriched lipid rafts in PM are critical for responding to extracellular stimuli via mediating signaling transduction and membrane trafficking (16), we hypothesized that ACAT1 might alter cholesterol distribution and subsequently affect cells’ responsive ability to the adipogenic stimuli, which contributed to the anti-adipogenic phenotype in ACAT1-deficient preadipocytes.

**Fig. 5:**
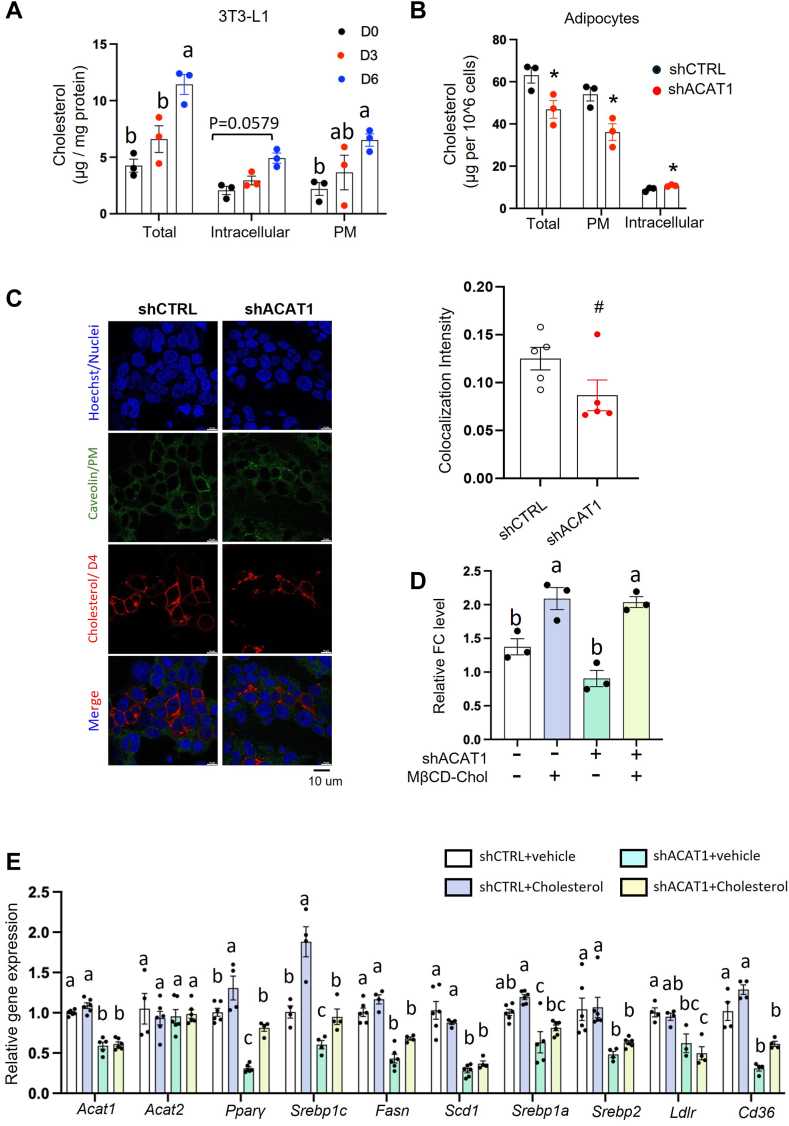
ACAT1 deficiency attenuated *Pparγ* transcription by reducing plasma membrane (PM)-cholesterol. A: 3T3-L1 differentiated adipocytes were collected at different time points during adipogenesis for the analysis of cholesterol in total (TC), PM, and intracellular compartment, respectively. B: TC, PM and intracellular cholesterol levels were quantified in adipocytes differentiated from shCTRL and shSOAT1 3T3-L1 preadipocytes. C: cholesterol distribution in shCTRL and shACAT1 of 293T cells as indicated by RFP-domain 4, PM and nuclei as indicated by caveolin-1 (green) and nuclei (blue), respectively, were captured by confocal microscopy. The colocalization of D4 signal with caveolin-1 was quantified in the right panel. shCTRL and shACAT1 preadipocytes were differentiated to mature adipocytes. MβCD-coated cholesterol (20 μg/ml) were applied for 15 min to replenish cholesterol to PM, followed by cellular cholesterol level quantification with the biochemical kit (D), and mRNA analysis with qPCR (E). Data is presented as Mean ± SEM (n = 3–5) and analyzed by student *t* test (B, C) or two-way ANOVA (A, D, E) followed by Tukey’s test. #*P* < 0.1, ∗*P* < 0.05. Different letters indicate statistically significant difference (*P* < 0.05).

To test this hypothesis, we first looked into the cholesterol distribution in PM and intracellular organelles. With the help of an oxidation-based assay (3), we observed that total cholesterol (TC) level and PM-localized FC significantly increased during adipogenesis, with a trending increment in intracellular FC (Fig. 5A). Knocking down ACAT1 attenuated the increment of PM-cholesterol during adipogenesis but accelerated intracellular-FC level as compared to shCTRL adipocytes (Fig. 5B). To further confirm these results, we transfected 293T cells with a recombinant RFP-tagged version of domain 4 (D4) expressing plasmid to visualize the FC in the cytosolic leaflets of membranes. D4 is a domain of Perfringolysin O and could bind to the accessible cholesterol without cytotoxicity (40). Consistently, compared to shCTRL cells, there were fewer D4-RFP signals distributed in the PM (Fig. 5C). Therefore, ACAT1 deficiency altered cholesterol distribution both in PM and intracellular segments. These data demonstrated that ACAT1 is an important player in regulating cholesterol redistribution during adipogenesis.

To further examine whether the reduction of PM-cholesterol in ACAT1-deficient adipocytes contributed to the attenuated PPARγ pathway, we replenished cholesterol with MβCD-coated cholesterol (Fig. 5D), which is well known to deliver cholesterol to PM directly (41, 42, 43, 44, 45). We found that MβCD-coated cholesterol successfully rescued the mRNA levels of adipogenic genes *Pparγ* and *Srebp1c* in shACAT1 preadipocytes in the presence of adipogenic cocktail (Fig. 5E). Collectively, ACAT1 deficiency reduced PM-cholesterol content, which subsequently contributed to the attenuated *Pparγ* transcription during the early stage of adipogenesis.

### ACAT1-deficiency inhibited SREBP2-mediated cholesterol uptake during adipogenesis

**Fig. 6:**
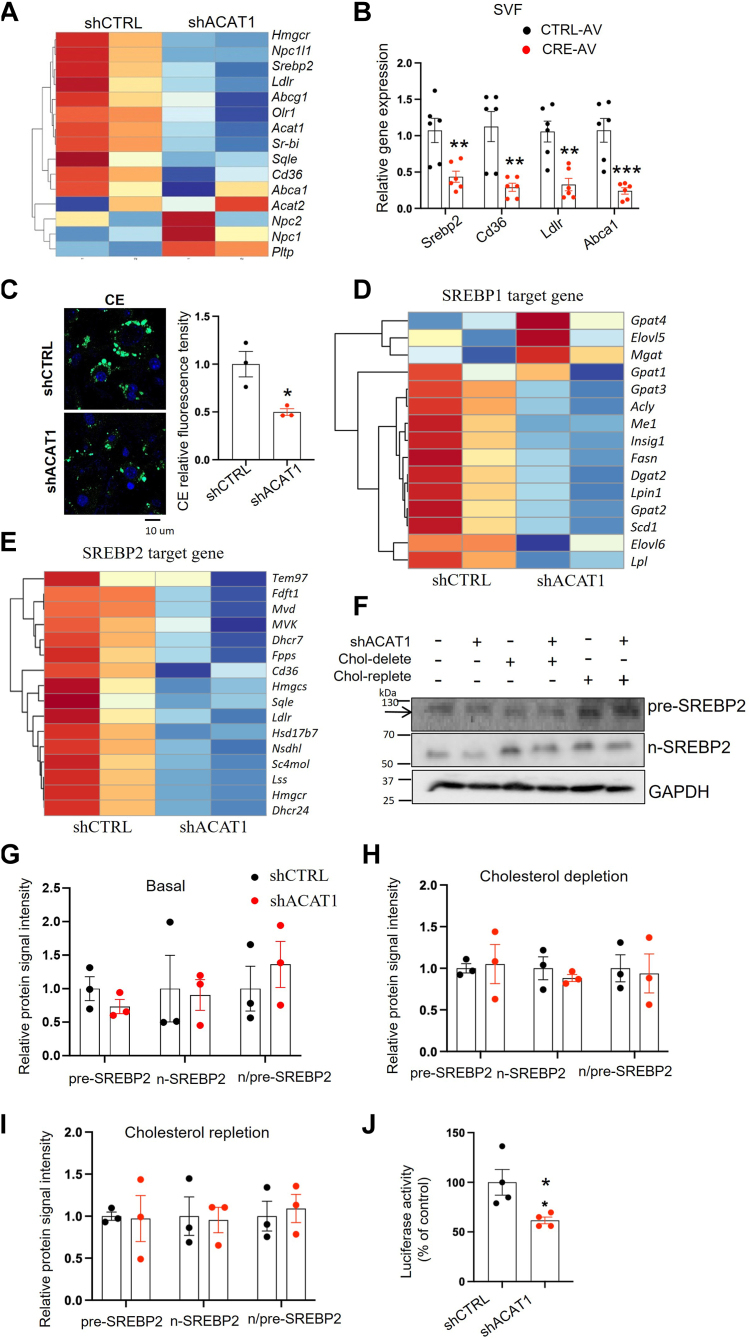
ACAT1 deficiency impairs SREBP2-mediated cholesterol uptake. A: the heatmap analysis of the cholesterol homeostasis associated genes were performed in shACAT1 versus shCTRL adipocytes after 3 days of differentiation. B: mRNA levels of genes involved in cholesterol metabolism in adipocytes differentiated from control (CTRL-AV) and ACAT1 (CRE-AV) deficient preadipocytes. C: 3T3-L1 adipocytes differentiated from shCTRL and shACAT1 preadipocytes were cultured with 0.5 μg/ml 22-NBD-cholesterol and examined under confocal microscopy for quantifying CE associated green fluorescence intensity. The heatmaps of SREBP1 (D) and SREBP2 (E) targeted genes were performed in shACAT1 versus shCTRL adipocytes after 3 days of differentiation. F: shCTRL and shACAT1 3T3-L1 cells under the conditions of cholesterol depletion and repletion were collected for the quantification of n-SREBP2 and pre-SREBP2 with WB, followed with the quantifications of band intensity with ImageJ (G–I). J: luciferase activity in shCTRL and shACAT1 adipocytes (3T3-L1) which were transfected with the SRE-luciferase plasmid. Data is presented as Mean ± SEM (n = 3–5) and analyzed by student *t* test. ∗*P* < 0.05, ∗∗*P* < 0.01, ∗∗∗*P* < 0.001.

The reason that resulted in lower PM-cholesterol and TC in shACAT1 adipocytes than shCTRL ones (Fig. 5B) could be either the increased cholesterol efflux or the decreased cholesterol uptake/synthesis. According to the RNAseq data (Fig. 6A), knocking down ACAT1 inhibited the upregulation of mRNA levels of several genes induced by adipogenic cocktail, and these genes are mainly involved in cholesterol uptake (*Ldlr*, *Cd36*) and de novo cholesterol synthesis (*Hmgcr*) (Fig. 6A, B). Complementarily, the dominant cholesterol efflux-related gene *Abca1* was also suppressed by ACAT1 knockdown during adipogenesis (Fig. 6A, B). Consistently, the synthetic ability of CE from FC was significantly reduced in shACAT1 adipocytes compared with shCTRL as indicated by the fluorescent intensity of 22-NBD-cholesterol (22-(N-(7-nitrobenz-2-oxa-1,3-diazol-4-yl)amino)-23,24-bisnor-5-cholen-3β-ol) (7) (Fig. 6C). Therefore, the inhibition of cholesterol uptake and synthesis may contribute to the attenuated cholesterol increment during adipogenesis in ACAT1-deficient cells.

Expression of genes responsible for cholesterol synthesis and uptake are largely regulated by SREBP2, while SREBP2 activation can be largely regulated by the presence of FC in ER (17). The activation of SREBPs requires the translocation of SREBPs from ER to the Golgi apparatus for the subsequent cleavage, resulting in the active N-terminal of SREBPs (n-SREBPs), which enters the nucleus and mediates the transcription of the downstream genes (46). This translocation is negatively regulated by the ER-localized cholesterol level (17). Our above data suggested a higher intracellular cholesterol level with ACAT1 deficiency (Fig. 5B), while our RNA-seq data revealed lower transcripts of both SREBP1 and 2, as well as their downstream targeted genes (Fig. 6D, E) in shACAT1 adipocytes than those in shCTRL ones (3 days after adipogenic stimulation). However, it is unknown whether the reduced expression of SREBPs’ downstream genes were predominantly due to the reduced expression of SREBPs, the reduced maturation of SREBPs, or the transcriptional regulatory ability of n-SREBP in the nucleus. Therefore, we first examined the pre-SREBP2 and n-SREBP2 protein levels in shCTRL and shACAT1 cells after four days of adipogenic stimulation. Interestingly, compared to shCTRL cells, although the mRNA level of SREBP2 was lower in ACAT1-deficient cells, pre-SREBP2, n-SREBP2, or n-SREBP2/pre-SREBP2 ratio were similar between shACAT1 and shCTRL cells under full medium, cholesterol depletion or cholesterol repletion conditions (Fig. 6F–I). Therefore, the protein level of SREBPs or the maturation of SREBPs may not be the predominant factors contributing to the low adipogenic ability in ACAT1-deficient cells. Next, we examined the transcriptional regulatory activity of n-SREBPs. Accordingly, we transfected adipocytes with the sterol regulatory elements (SRE)-luciferase plasmid and found that SRE luciferase activity was significantly decreased in shACAT1 cells compared with shCTRL ones (Fig. 6J). This is consistent with the lower transcription of SREBP2’s downstream genes (*Ldlr, Cd36*) in shACAT1 adipocytes than shCTRL ones (Fig. 6B). Thus, these data suggested that either the transportation of n-SREBP2 to the nucleus or the transcriptional regulatory ability of n-SREBPs might be the dominant factor contributing to the reduced cholesterol uptake in ACAT1-deficient cells. However, it remains unclear whether this reduction in n-SREBP2 activity significantly contributes to adipogenesis or not. Collectively, ACAT1-deficiency inhibited SREBP2-mediated cholesterol uptake during adipogenesis.

### ACAT1-mediated cholesterol homeostasis is responsible for maintaining adipogenic ability

**Fig. 7:**
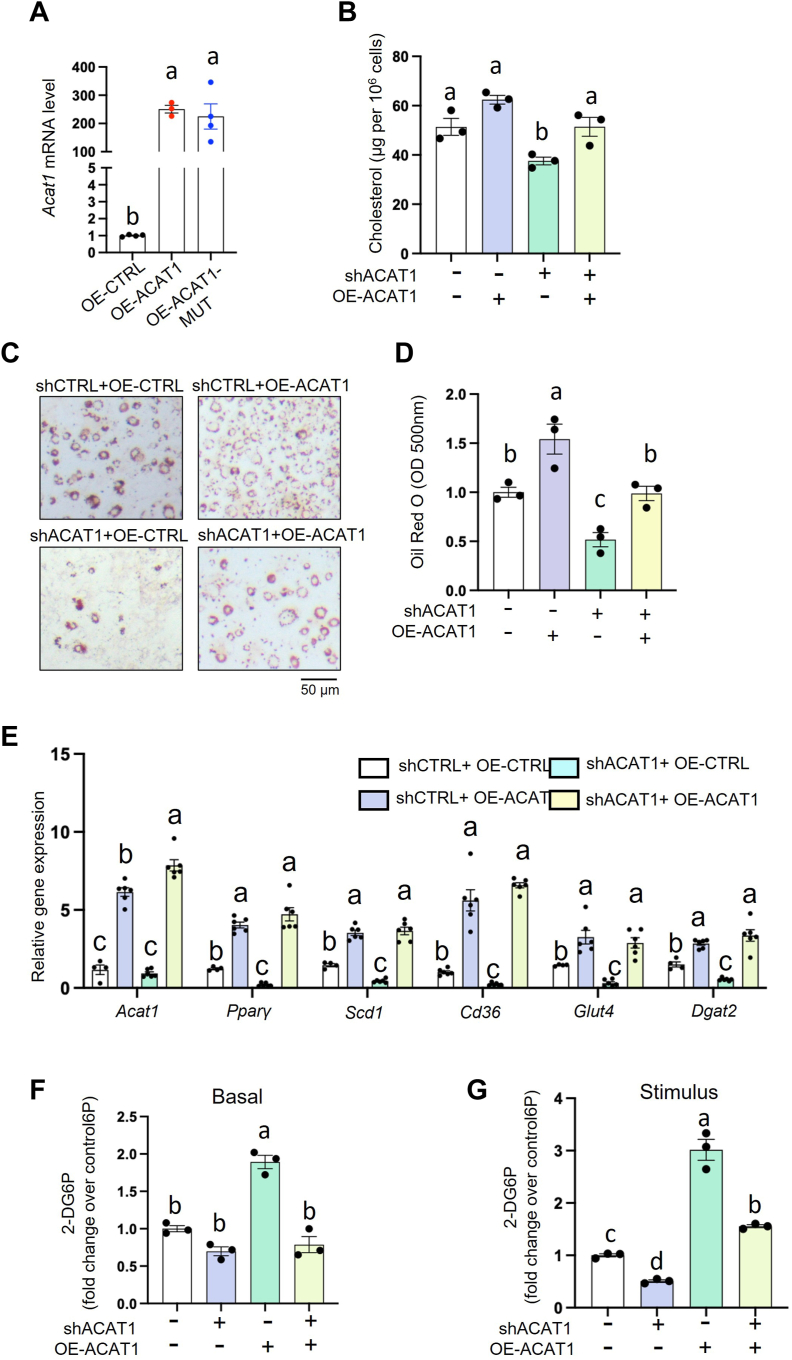

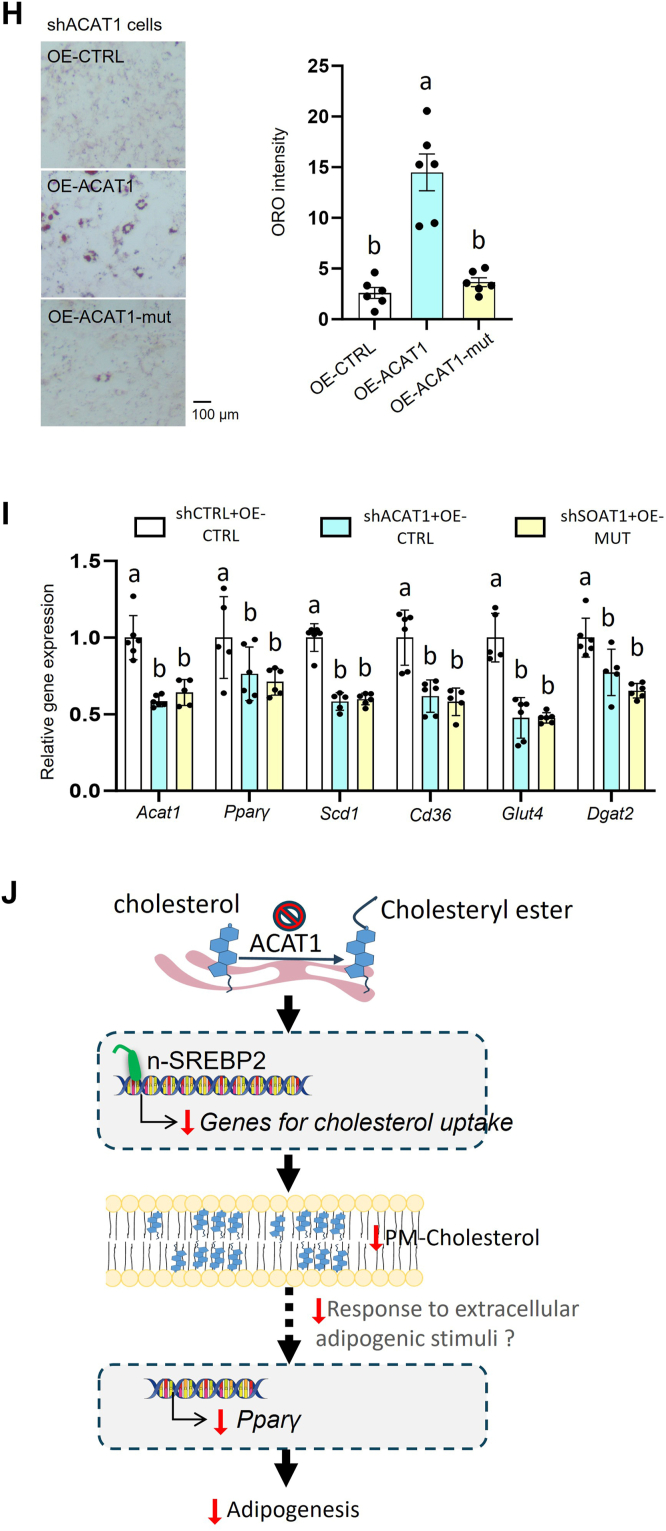
ACAT1 helps maintain adipogenic ability by mediating cholesterol homeostasis. A: *Acat1* mRNA level in 3T3-L1 preadipocytes with ACAT1 or ACAT1-mutant overexpression. In shCTRL and shACAT1 preadipocytes, we overexpressed human catalytically functional ACAT1 and induced adipogenesis. Then the total cellular cholesterol level (B), lipid level with ORO staining (C, D), and mRNA level with qPCR (E) were quantified. shCTRL and shACAT1 preadipocytes were differentiated for 3–4 days and glucose uptake ability was measured with 2-DG6P in the absence (F) or the presence (G) of insulin. Overexpressing ACAT1 and catalytic-dead ACAT1-mutant in shACAT1 preadipocytes, and subsequently differentiate for 6 days. Lipid staining with ORO (H) and gene expressions with q-PCR (I) were quantified. (J) schematic illustration the absence of ACAT1 leads to impaired adipogenesis in adipocytes. Data is presented as Mean ± SEM (n = 3–5) and analyzed by two-way ANOVA followed by Tukey’s test. Different letters indicate statistically significant difference (*P* < 0.05).

ACAT1 is considered to esterify FC to CE on the ER, regulating FC and CE pools. To further examine whether ACAT1 participates in adipogenesis via regulating cholesterol homeostasis, we overexpressed catalytically functional ACAT1 and catalytically dysfunctional ACAT1. Adenovirus-mediated overexpression of human-*Acat1* (OE-ACAT1) was employed to increase *Acat1* mRNA by 200–250-fold in preadipocytes (Fig. 7A). As expected, ACAT1 knockdown attenuated the increment of TC induced by the adipogenic cocktail during adipogenesis, while this attenuation was rescued by OE-ACAT1 (Fig. 7B). Furthermore, overexpressing *Acat1* rescued the attenuated adipogenesis in shACAT1 cells, as indicated by the rescued lipid content (Fig. 7C, D) with increased mRNA levels of genes involved in adipogenesis (*Pparγ*) and lipid synthesis (*Dgat2, Glut4, Cd36, Scd1*) (Fig. 7E). Moreover, given that glucose uptake is critical for adipogenesis in vitro, we overexpressed ACAT1 in shCTRL and shACAT1 preadipocytes, respectively. After four days’ adipogenic induction, glucose uptake ability was similar between shCTRL and shACAT1 cells in the absence of insulin (Fig. 7F), while overexpressing ACAT1 fully rescued the 50% reduction of glucose uptake ability in shACAT1 cells in the presence of insulin (Fig. 7G). In contrast, overexpressing the catalytic dead mutant *Acat1* (*Acat1-H460A*) could not rescue the impaired adipogenic ability in shACAT1 preadipocytes, as indicated by lipid accumulation (Fig. 7H) and adipogenic gene expression (Fig. 7I). Collectively, our above data suggested that ACAT1’s enzymatic function (i.e. ACAT1-mediated cholesterol homeostasis) is responsible for maintaining preadipocytes’ adipogenic ability (Fig. 7J).

## Discussion

WAT is the primary storage depot for TG and it contains a high amount of FC in our body (47). Several studies have reported that disrupting cholesterol homeostasis impairs adipocyte function (48). However, the role of cholesterol esterification in regulating adipogenesis remains understudied. In our previous study, knocking down ACAT1, which esterifies cholesterol, could reduce lipogenic gene expression and suppress adipogenesis in murine 3T3-L1 cells (18). However, how ACAT1 regulates adipogenesis remains unclear. Here, we identified ACAT1 as the dominant isoform in WAT of humans and mice and demonstrated that ACAT1’s contribution in maintaining adipogenic ability is via regulating cholesterol distribution and PPARγ-signaling pathway at the early stage of adipogenesis.

ACAT1 is widely expressed in various tissues of adult mice (49) and predominantly in adrenal glands in humans (50). However, the expression of ACAT1 in WAT is unclear. Here, by re-analyzing the RNA-seq data of human WAT from other research groups (27) and isolating mature adipocytes from mouse WAT, we found that ACAT1 mRNA and protein levels were positively associated with adiposity in either mice or human samples, while ACAT2 protein level was undetectable (Fig. 1D). This absence of ACAT2 protein band may be due to either the low expression level or its easily degradable property, as ACAT2 holds fewer transmembrane domains than ACAT1 in ER (6, 51). Collectively, ACAT1 is the dominant isoform regulating cholesterol esterification in both human and murine WAT. Thus, we focused on ACAT1 in the later investigations. Notably, in murine samples, the knockdown of either ACAT1 or ACAT2 could suppress adipogenesis (18), suggesting the non-negligible roles of either isoform of ACATs. While the functional overlap of ACAT1 and ACAT2 in adipocytes remains uncertain, caution may be required regarding the potential compensatory effect between ACAT1 and ACAT2 when examining the role of ACATs in adipocytes using murine samples. Furthermore, it is important to acknowledge the differences in expression patterns in WAT between humans and mice.

We previously reported that the attenuated adipogenesis in ACAT1-deficient preadipocytes is partially attributed to the reduced transcription of *Srebp1* and its downstream lipogenic gene expression (18), which occurs at the end of the adipogenic cascade. However, when looking at the upstream, ACAT1 knockdown altered adipogenic marker genes significantly at the early stage of adipogenesis (Figs. 3E and 4A), especially reducing the transcriptions of adipogenic master gene *Pparγ*. As expected, rescuing PPARγ transcriptional activity by a chemical agonist or nutritional ligand could rescue the adipogenic ability in shACAT1 preadipocytes (Fig. 4B–G). These data demonstrated that ACAT1’s contribution in maintaining adipogenic ability is at the early stage of adipogenesis and is, at least partially, PPARγ dependent.

Our results demonstrated that ACAT1 regulated cholesterol quantity and distribution during adipogenesis in vitro. The knockdown of ACAT1 resulted in a decrease in cholesterol levels in the PM and an increase in intracellular compartments compared to control cells during adipogenesis, respectively (Fig. 5A, B). However, ACAT1’s role in regulating PM-cholesterol varies among different cell types. Inhibiting ACAT1 in CD8^+^ T cells led to the increment of PM-cholesterol and subsequently enhanced T-cell cytotoxicity towards melanoma tumors (3). In contrast, activating ACAT1 in macrophages led to decreased PM-cholesterol and further underpins immunological activities (52). In adipocytes, PM-cholesterol is crucial for maintaining the cell responsiveness to extracellular stimuli. We found that preadipocytes’ response to the adipogenic cocktail was impaired in ACAT1-deficient cells but rescued by β-cyclodextrin cholesterol-loading (Fig. 5D, E). Similarly, a progressive depletion of cholesterol in 3T3-L1 adipocytes attenuated glucose uptake under insulin stimulation (53). Supportively, long-chain fatty acid uptake in adipocytes was suppressed by β-cyclodextrin-mediated PM-sterol depletion but rescued by cholesterol repletion (54). Collectively, ACAT1 is critical for maintaining optimal cholesterol distribution in the PM of preadipocytes. However, whether ACAT1-mediated regulation of cholesterol homeostasis is important for preserving the responsiveness of preadipocytes to extracellular stimuli, which is a key factor influencing the adipogenic differentiation process, requires further investigations. Precise measurement of cholesterol and lipid rafts in the PM, as well as the cholesterol trafficking between intracellular organelles require the development of better biochemical tools and imaging techniques to provide direct evidence.

Although ACAT1 deficiency altered cholesterol distribution and increased intracellular cholesterol levels when compared to control ones (Fig. 5B, C), the detailed intracellular distribution of cholesterol is unknown. We analyzed the RNA-seq data and depicted the main genes that are responsible for cholesterol trafficking (supplemental Table S1). Firstly, the upregulation of *Npc1* and *Npc2* during adipogenesis was more pronounced in shACAT1 adipocytes than in shCTRL ones (Fig. 6A). NPC2 is a soluble protein located in the lysosome that aids in delivering cholesterol to NPC1, facilitating cholesterol transport from endosomes/lysosomes to other cellular compartments (55). Consistently, OSBPL5, a cholesterol sensor facilitating the movement of LDL-derived cholesterol from the lysosome to the ER (56, 57), decreased during adipogenesis (supplemental Fig. S2A), while this decline was inhibited by ACAT1 knockdown (supplemental Fig. S2B). Thus, ACAT1 deficiency may enhance cholesterol trafficking out of endosomes/lysosomes. Secondly, GRAMD1B has been shown to participate in the non-vesicular transport of cholesterol, facilitating the movement of accessible PM cholesterol to the ER (58). In our study, we observed an increase in *Gramd1b* mRNA during adipogenesis (supplemental Fig. S2A). However, the knockdown of ACAT1 hindered this increase (supplemental Fig. S2C), suggesting that ACAT1 deficiency may reduce GRAMD1B-mediated cholesterol transport from PM to ER, which is speculated to be due to the elevated ratio of ER/PM cholesterol. Supportively, caveolin-1, a protein typically located in cholesterol-riched caveolae, was remarkably lower with ACAT1 deficiency (supplemental Fig. S2D), suggesting a lower PM-cholesterol level with ACAT1-deficiency. Collectively, ACAT1 deficiency may alter cholesterol trafficking among intracellular organelles, especially involving endosomes, lysosomes, PM, and ER. However, the detailed cholesterol trafficking between the intracellular organelles requires the development of reliable and sensitive techniques.

Consistent with that adipogenesis is hindered by ACAT1 knockdown in vitro, reduced WAT mass was found in the global ACAT1-knockout (KO) mice as compared to the WT mice, when fed with chow diet. However, we cannot exclude the contribution of ACAT1 from non-preadipocytes to this phenotype in KO mice. Further studies using AdipoChaser mice are needed to provide direct evidence. Given that the attenuation of adipogenic ability in ACAT1 deficient preadipocytes was PPARγ dependent, supplementing PPARγ ligand OA is a nutritional intervention to upregulate the transcriptional activity of PPARγ and thus restore the adipogenic ability in ACAT1-deficient preadipocytes (Fig. 4E–G). Notably, from a metabolite perspective, supplementation with OA alone was able to restore adipogenesis in shACAT1 preadipocytes (Fig. 4E–G) to a level comparable to that seen with cholesterol replenishment (Fig. 5D, E) and ACAT1 overexpression (Fig. 7B–E). Therefore, the primary downstream mediators of ACAT1's impact on adipogenesis may include both oleic acids and cholesterol. Therefore, the nutrient-gene interaction may partially explain the remained WAT development in these KO mice. Interestingly, in the high-fat diet-induced obese mouse model, global knocking out ACAT1 showed either higher or lower body weight than WT mice from studies conducted in China and the USA (19, 59), respectively, leaving ACAT1’s physiological role inconclusive. Based on our findings, we speculated that the different amounts of dietary oleic acid might be a possible reason contributing to these opposite phenotypes.

In summary, our results demonstrated that ACAT1 is the main isoform related to adiposity in both human and murine models and that ACAT1 helps maintain the adipogenic ability via regulating PM-cholesterol level and ultimately regulating the PPARγ signaling pathway. Regardless of the limited CE quantity in adipocytes, cholesterol esterification is important in maintaining the adipogenic ability.

## Data availability

The RNA sequencing data reported in this paper were deposited in the NCBI’s Sequence Read Archive (BioProject ID: PRJNA1061348).

## Supplemental data

This article contains supplemental data.

## Conflict of interest

The authors declare that they have no conflicts of interest with the contents of this article.
