# Supplementary material for: ACAT1/SOAT1 maintains adipogenic ability in preadipocytes by regulating cholesterol homeostasis
Source: J Lipid Res. 2024 Oct 30;65(12):100680. doi: 10.1016/j.jlr.2024.100680 (PMC11638590; doi:10.1016/j.jlr.2024.100680)
Supplement: Supplementary information [file mmc1.docx]

Supplementary materials

Figure S1.


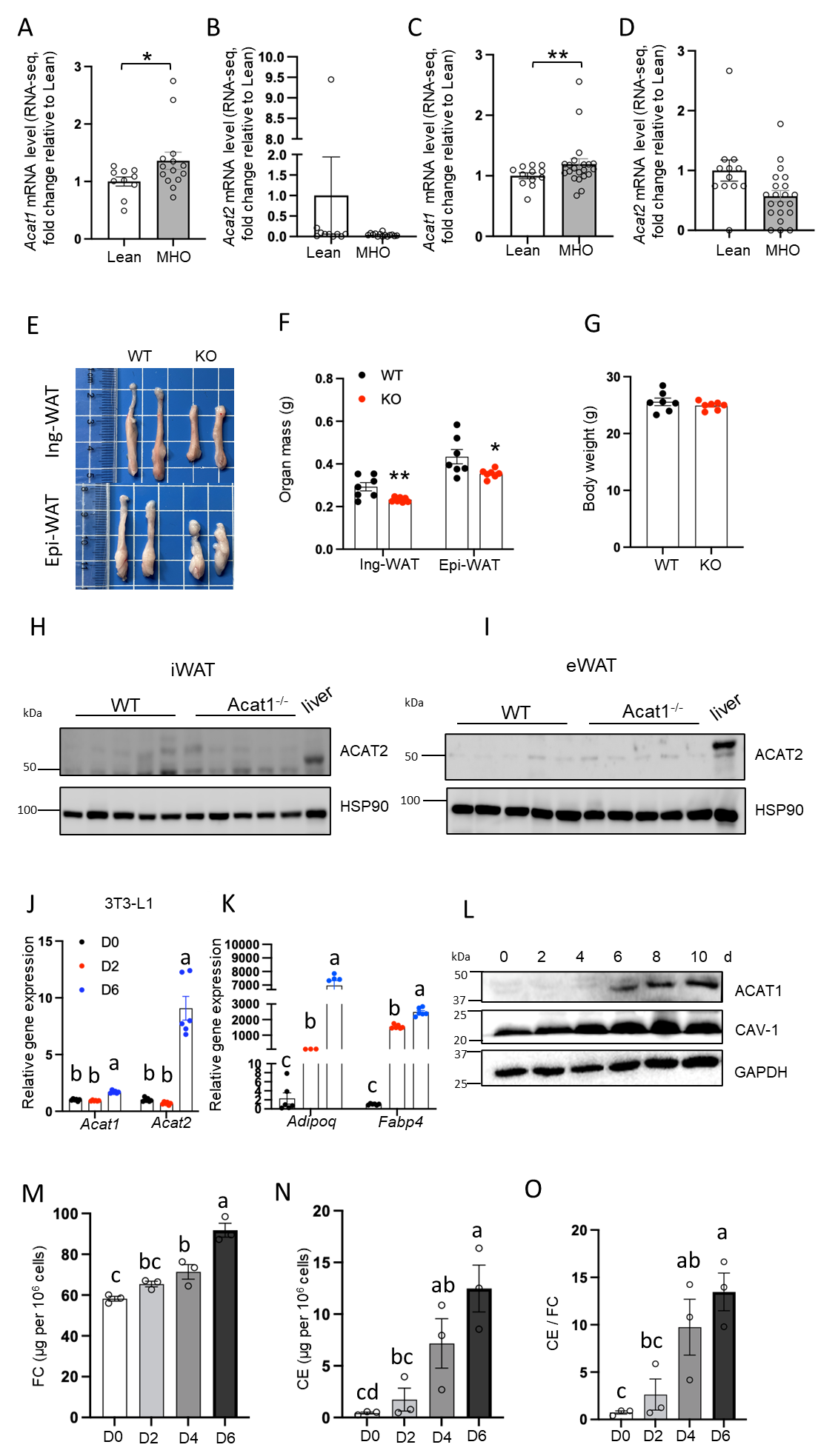


**Supplementary Figure 1. ACAT1 is associated with adiposity.** ACAT1 (A) and ACAT2 (B) expression in subcutaneous abdominal adipose tissue in the lean (n=10) and metabolic healthy obese (MHO; n=14) subjects. ACAT1 (C) and ACAT2 (D) expression in subcutaneous abdominal adipose tissue in the lean (n=12) and metabolic healthy obese (MHO; n=21) subjects. Wild type (WT) and global ACAT1-knockout (KO) male mice were fed with chow diet and sacrificed at 8 weeks old (n=7) for the analysis of WAT morphology (E), WAT mass (F), and body weight (G). ACAT2 protein levels in Ing-WAT (H) and Epi-WAT (I) of WT and ACAT1-KO mice by western-blot (WB). 3T3-L1 preadipocytes were differentiated and collected at different time points during adipogenesis for the analysis of indicated genes using qPCR (J, K), proteins using WB (L), and the quantification of FC (M), CE (N), as well as CE : FC (O). Data is presented as Mean ± SEM (n = 3-5) and analyzed by student t-test (A-D, F) or two-way ANOVA followed by Tukey’s test (J-K, M-O). * *p* < 0.05, ** *p* < 0.01. Different letters indicate statistically significant difference (*p* < 0.05).

Figure S2.

**
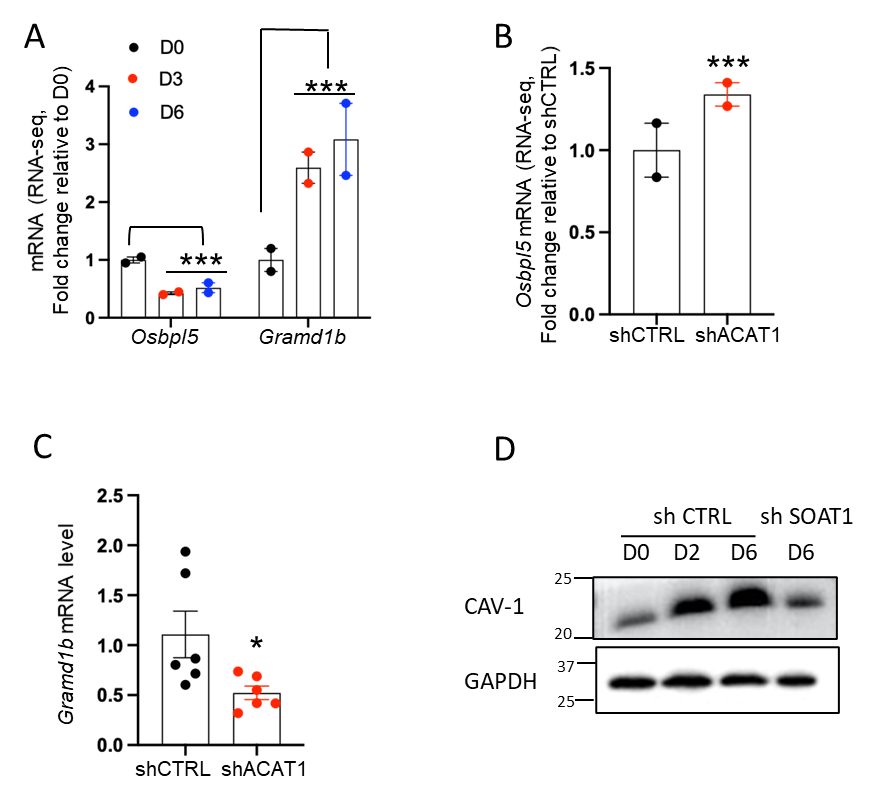
**

**Supplementary Figure 2. ACAT1 deficiency alters the expression of genes involved in cholesterol trafficking during adipogenesis.**

(A) 3T3-L1 differentiated adipocytes were collected at different time points during adipogenesis for the analysis of indicated genes using qPCR. (B) Osbpl5 mRNA level in 3T3-L1 differentiated shCTRL and shACAT1 adipocytes from RNA-seq data. (C) Gramd1b mRNA level in 3T3-L1 differentiated shCTRL and shACAT1 adipocytes by using qPCR. (D) Caveolin-1 (CAV-1) protein levels in different differentiation time of shCTRL and shSOAT1 by WB. Data is presented as Mean ± SEM (n = 3-5) and analyzed by student t-test. * *p* < 0.05, *** *p* < 0.001.

Table S1 Summary of cholesterol trafficking gene

| Cholesterol Trafficking direction | Related gene name | mRNA Changes during day 0-3 | mRNA Changes during day 3-6 | Effect of SOAT1 deficiency during day 0-3 | Effect of SOAT1 deficiency during day 3-6 | references |
| --- | --- | --- | --- | --- | --- | --- |
| Transport out of lysosome | NPC1 | Significantly decreased | Significantly increased | Significantly decreased | Significantly increased | [1] |
| Transport out of lysosome | NPC2 | Significantly decreased | Significantly increased | Trend to decrease | Significantly increased | [1] |
| Transport out of lysosome | LAMP2 | Significantly decreased | Significantly increased | Significantly decreased | Significantly increased | [2] |
| Lysosome to ER | OSBPL1a | No difference | Significantly decreased | No difference | No difference | [3] |
| Lysosome to ER | OSBPL5 | Significantly decreased | No difference | Significantly decreased | Trend to increase | [4] |
| Lysosome to PM | OSBPL2 | No difference | Significantly increased | No difference | No difference | [5] |
| Lysosome to Golgi | STARD5 | No difference | Significantly increased | No difference | No difference | [6] |
| Between ER and PM | OSBPL8 | No difference | Significantly increased | No difference | Trend to increase | [7, 8] |
| PM to ER | GRAMD1b | Significantly increased | No difference | Significantly increased | No difference | [9] |
| ER to lysosome | STARD3 | No difference | No difference | No difference | No difference | [10] |

Table S2 Plasmid sequence

| Plasmid | Sequence |
| --- | --- |
| D4-RFP | GGTGGAGGCGGTTCAAAGGGAAAAATAAACTTAGATCATAGTGGAGCCTATGTTGCACAGTTTGAAGTAGCATGGGATGAAGTTTCAGCgcTGACAAAGAAGGAAATGAAGTTTTAACTCATAAAACATGGGATGGAAATTATCAATGGtggAAAACAGCTCACTATTCAACAGTAATACCTCTTGAAGCCAATGCAAGAAATATAAGAATAAAGGCAAGAGAGTGTACAGGTCTTTGGtggTGGGAATGGTGGAGAGATGTTATAAGTGAATATGATGTTCCATTAACAAATAATATAAATGTTTCAATATGGGGAACTACTTTATACCCTGGATCTAGTATTACTTACAATTAA |

Table S3 Real time PCR primers

| Gene name | Forward primer | Revere primer |
| --- | --- | --- |
| GAPDH | AAGAAGGTGGTGAAGCAGGCATC | CGAAGGTGGAAGAGTGGGAGTTG |
| β-actin | AGATGACCCAGATCATGTTTGAGA | CACAGCCTGGATGGCTACGT |
| SOAT1 | TCGCACTCCTCATCCTATTCG | GTACCAGCCTTCCTTCATCGAT |
| SOAT2 | AGACTTGGTGCAATGGACTCGAC | CATAGGGCCCGATCCAACAG |
| ADIPOQ | GGAACTTGTGCAGGTTGGAT | GCTTCTCCAGGCTCTCCTTT |
| FABP4 | GATGAAATCACCGCAGACGACA | ATTGTGGTCGACTTTCCATCCC |
| LEPTIN | CACACACGCAGTCGGTATCC | AGCCCAGGAATGAAGTCCAA |
| PPARγ | ACCTCTGCTGGGGATCTGAA | TGGCATTGTGAGACATCCCC |
| DGAT2 | GCCGTGTGGCGCTACTTC | GTGGTCAGCAGGTTGTGTGTCT |
| SCD1 | GGTGATGTTCCAGAGGAGGTACTAC | AGCGTGGGCAGGATGAAG |
| SREBP1c | TGGGAACCCAGTGGATC | GCAGCATCAGAACAGCTATTTA |
| GLUT4 | GGCTTTGTGGCCTTCTTTGAG | GACCCATAGCATCCGCAACAT |
| CD36 | GTTCTTCCAGCCAATGCCTTT | ATGTCTAGCACACCATAAGATGTACAGTT |
| ACC | GTCCCCAGGGATGAACCAATA | GCCATGCTCAACCAAAGTAGC |
| GPAT3 | AGTGAGCTGGTGCATCTGACCT | GATTGGCGACACAGATACCTCC |
| AGPAT2 | TTTGAGGTCAGCGGACAGAA | AGGATGCTCTGGTGATTAGAGATGA |
| FASN | CTACTGCGATTCTCCTGGC | GATTTCTGGGACTTTGTTTCCTG |
| SREBP2 | TGTGAACCTGGCCGAGTGT | CGCTGTCAGGTGGATCTCAA |
| LDLR | AGGCTGTGGGCTCCATAGG | TGCGGTCCAGGGTCATCT |
| LPL | GCGTAGCAGGAAGTCTGACCAA | AGCGTCATCAGGAGAAAGGCGA |
| ABCA1 | AGTTTCGGTATGGCGGGTTT | AGCATGCCAGCCCTTGTTAT |
| NPC1 | GGGATGCCCGTGCCTGCAAT | CTGGCAGCTACATGGCCCCG |
| NPC2 | TATCTTGTGACTGCTCGG | CTGGTAAAGGTGATGTTGA |
| CD146 | CGAGGCAGAAAGTAACCAGGAC | GTCTCACGTTGTTTAGCTGGAGG |

Reference:

1. Storch, J. and Z. Xu, *Niemann-Pick C2 (NPC2) and intracellular cholesterol trafficking.* Biochim Biophys Acta, 2009. **1791**(7): p. 671-8.

2. Xiong, Q. and Y. Rikihisa, *Subversion of NPC1 pathway of cholesterol transport by Anaplasma phagocytophilum.* Cell Microbiol, 2012. **14**(4): p. 560-76.

3. Zhao, K. and N.D. Ridgway, *Oxysterol-Binding Protein-Related Protein 1L Regulates Cholesterol Egress from the Endo-Lysosomal System.* Cell Rep, 2017. **19**(9): p. 1807-1818.

4. Du, X., et al., *A role for oxysterol-binding protein-related protein 5 in endosomal cholesterol trafficking.* J Cell Biol, 2011. **192**(1): p. 121-35.

5. Olkkonen, V.M. and E. Ikonen, *Cholesterol transport in the late endocytic pathway: Roles of ORP family proteins.* J Steroid Biochem Mol Biol, 2022. **216**: p. 106040.

6. Rodriguez-Agudo, D., et al., *StarD5: an ER stress protein regulates plasma membrane and intracellular cholesterol homeostasis.* J Lipid Res, 2019. **60**(6): p. 1087-1098.

7. Chung, J., et al., *INTRACELLULAR TRANSPORT. PI4P/phosphatidylserine countertransport at ORP5- and ORP8-mediated ER-plasma membrane contacts.* Science, 2015. **349**(6246): p. 428-32.

8. Kennelly, J.P. and P. Tontonoz, *Cholesterol Transport to the Endoplasmic Reticulum.* Cold Spring Harb Perspect Biol, 2023. **15**(2).

9. Naito, T., et al., *Movement of accessible plasma membrane cholesterol by the GRAMD1 lipid transfer protein complex.* Elife, 2019. **8**.

10. Wilhelm, L.P., et al., *STARD3 mediates endoplasmic reticulum-to-endosome cholesterol transport at membrane contact sites.* EMBO J, 2017. **36**(10): p. 1412-1433.
